# Extratumoral Heme Oxygenase-1 (HO-1) Expressing Macrophages Likely Promote Primary and Metastatic Prostate Tumor Growth

**DOI:** 10.1371/journal.pone.0157280

**Published:** 2016-06-09

**Authors:** Sofia Halin Bergström, Maria Nilsson, Hanibal Adamo, Elin Thysell, Emma Jernberg, Pär Stattin, Anders Widmark, Pernilla Wikström, Anders Bergh

**Affiliations:** 1 Department of Medical Biosciences, Pathology, Umeå University, Umeå, Sweden; 2 Department of Surgical and Perioperative Sciences, Urology, Umeå University, Umeå, Sweden; 3 Department of Radiation Sciences, Oncology, Umeå University, Umeå, Sweden; University of Pécs Medical School, HUNGARY

## Abstract

Aggressive tumors induce tumor-supporting changes in the benign parts of the prostate. One factor that has increased expression outside prostate tumors is hemoxygenase-1 (HO-1). To investigate HO-1 expression in more detail, we analyzed samples of tumor tissue and peritumoral normal prostate tissue from rats carrying cancers with different metastatic capacity, and human prostate cancer tissue samples from primary tumors and bone metastases. In rat prostate tumor samples, immunohistochemistry and quantitative RT-PCR showed that the main site of HO-1 synthesis was HO-1^+^ macrophages that accumulated in the tumor-bearing organ, and at the tumor-invasive front. Small metastatic tumors were considerably more effective in attracting HO-1^+^ macrophages than larger non-metastatic ones. In clinical samples, accumulation of HO-1^+^ macrophages was seen at the tumor invasive front, almost exclusively in high-grade tumors, and it correlated with the presence of bone metastases. HO-1^+^ macrophages, located at the tumor invasive front, were more abundant in bone metastases than in primary tumors. HO-1 expression in bone metastases was variable, and positively correlated with the expression of macrophage markers but negatively correlated with androgen receptor expression, suggesting that elevated HO-1 could be a marker for a subgroup of bone metastases. Together with another recent observation showing that selective knockout of HO-1 in macrophages reduced prostate tumor growth and metastatic capacity in animals, the results of this study suggest that extratumoral HO-1^+^ macrophages may have an important role in prostate cancer.

## Introduction

In order to grow and spread, cancers need to instruct adjacent cells as well as remote organs to cooperate. For example, malignant cells signal to the surrounding cells and reshape them into a tumor-promoting stroma [1, 2]. In addition, tumors recruit cells from the bone marrow–for example, stem cells, inflammatory cells, and cancer-associated fibroblasts (CAFs)–that contribute to tumor formation and growth [1, 2]. Moreover, tumors send signals to pre-metastatic niches to prepare the soil for subsequent metastatic colonization [1, 2]. One additional, often neglected, site that also adapts to support tumor growth is the tumor-bearing organ [3].

To explore how the surrounding prostate tissue adapts to the presence of a tumor, we implanted rat prostate cancer cells into the prostates of syngenic and immune-competent rats [3–8]. We found that tumor growth resulted in several modifications in the tumor-bearing organ–for example, growth of the vasculature and alterations in the extracellular matrix [6, 8, 9]. These changes were found to be partially mediated by accumulating inflammatory cells such as macrophages and mast cells [10–12]. Importantly, already when small, fast-growing and metastatic tumors induced more pronounced changes than larger, slow-growing and non-metastatic tumors [5].

In patients, similar changes have been seen in the tumor-bearing prostate and the magnitude of these alterations was found to be related to tumor aggressiveness and patient outcome (for reviews see [3, 5, 13]). As the surrounding normal prostate tissue apparently adapts to the needs of the growing tumor, we have proposed that this tissue response should be named TINT: tumor instructed normal tissue [3]. TINT is an adaptive response induced by the tumor in histologically normal-appearing epithelium and stroma. TINT is therefore different from field cancerization, which is defined as premalignant genetic changes that are presumably due to a cancerogenic agent that has affected the entire organ [5, 14, 15].

One gene that shows highly increased expression in rat prostate TINT is hemoxygenase-1 (HO-1) [4]. HO-1 is an inducible enzyme that converts heme into carbon monoxide (CO), iron (Fe), and biliverdin. HO-1 and its by-products function as cytoprotectants, antioxidants, and anti-inflammatory, anti-apoptotic, anti-proliferative, and immune-modulatory factors [16, 17]. HO-1, which is generally expressed in subtypes of macrophages, is involved in macrophage maturation and polarization towards the tumor-stimulating M2 phenotype [16, 18, 19]. HO-1 can also be pro-inflammatory, and HO-1 expressing macrophages are central to the pathogenesis of insulin resistance and metabolic syndrome [20]. HO-1 may also have functions unrelated to heme degradation [17, 21], and when localized to the nucleus it may affect transcription [21].

In prostate cancer, HO-1 has been detected in tumor epithelial cells, including the nucleus, and high HO-1 levels were found to be associated with high Gleason grade and poor outcome [22–28]. HO-1 has also been shown to be increased in the serum of prostate cancer patients, but the levels were not related to tumor Gleason score or serum PSA [23]. Inhibition of HO-1 function in tumor epithelial cells, through gene silencing or by blocking of the enzyme activity with a systemic HO-1 inhibitor, has been shown to reduce prostate tumor growth [22]. Conversely, overexpression of HO-1 in prostate tumor epithelial cells also reduced tumor growth [26, 29–32]. HO-1 has also been detected in tumor-infiltrating macrophages, and specific knockout of HO-1 in these cells inhibited prostate tumor growth [33]. The functional roles and sites of HO-1 synthesis and action in prostate cancer are thus somewhat unclear.

Several factors that were found to be upregulated in rat TINT, such as lysyl oxidase [4, 8, 34], hyaluronic acid [9], mast cells [12], subsets of macrophages [5, 11, 35, 36], and blood vessels [6, 37] were also increased in human prostate TINT, and these were related to tumor aggressiveness and patient outcome in a watchful waiting cohort [3, 5, 13]. We therefore hypothesized that HO-1 might belong to this family of TINT factors that promote tumor growth. To investigate the possible role of TINT-derived HO-1 in prostate tumors, we examined HO-1 expression both in the Dunning rat prostate cancer model (which included tumors with different aggressiveness) and in patient samples from primary prostate cancers and bone metastases. We found that HO-1 was mainly expressed in infiltrating macrophages at the invasive front and in the non-malignant prostate tissue (TINT) of aggressive rat prostate tumors. In patient samples, HO-1^+^ macrophages were found at the invasive front of high-grade tumors while tumor epithelial HO-1 expression was low. HO-1 expression was higher in bone metastases than in primary tumors and was mainly expressed by macrophages. Taken together with the results of previous studies, this suggests that extratumoral HO-1 expressing macrophages are likely involved in prostate cancer aggressiveness and metastatic capacity, and that their role in bone metastases in particular should be explored in more detail.

## Materials and Methods

### Ethics statement

Patient material 1 consisted of tissue from transurethral resection of the prostate between 1975 and 1991. This material was collected and stored as part of standard health care procedures and according to the Swedish regulations at a time when informed consent was not required. The research ethical committee at Umeå University Hospital (Regional Ethical Review Board in Umeå) approved the study (permit number 02–283) and waived the need for consent. Patient information was anonymized and de-identified prior to analysis.

Patient material 2 consisted of non-malignant prostate, malignant prostate, and bone metastases removed during surgical treatments for localized and metastatic prostate cancer. This part of the study was approved by the Regional Ethical Review Board in Umeå (permit number 03–185). All patients provided written informed consent.

For the animal studies, all animal work was carried out in accordance with protocols approved by the Umeå Ethical Committee for animal research (permit number A110-12). Adult Copenhagen rats (300–400 g) were housed in a well-ventilated room under controlled light (12 h light/ 12 h dark), and temperature (25°C), and monitored on a daily basis by an experienced animal caretaker. Food and water were available ad libitum. Animals were anesthetized with intraperitoneal injections of Ketamine (75 mg/kg) and Medetomidine (0.5 mg/kg) before tumor cell injection and at sacrifice. In this study, all the tumors were small and were still surrounded by normal prostate tissue, and did not give rise to any symptoms in the animals. All animals included in the study survived to the endpoint of the experiment. If the animals would have developed signs of severe illness (such as low appetite, weight loss, urinary problems, lethargy, and shortness of breath) they would have been euthanized immediately.

### Orthotopic implantation of rat prostate tumor cells

Dunning G, AT-1, and MatLyLu rat prostate tumor cells (ECACC, Sigma Aldrich, Stockholm, Sweden) were grown in RPMI 1640+GlutaMAX (Gibco, Thermo Scientific, Waltham, MA) supplemented with 10% fetal bovine serum (Gibco) and 250 nM dexamethasone (Sigma) as previously described [38]. For morphological analysis, AT-1 (2 x 10^3^ cells in 10 μl of RPMI 1640), MatLyLu (2 x 10^3^ cells in 10 μl of RPMI 1640), or G cells (2 x 10^3^ cells or 2 x 10^5^ cells in 10 μl of RPMI 1640) were carefully injected into one lobe of the ventral prostate of adult Copenhagen rats (Charles River, Sulzfeld, Germany) as previously described [4]. Rats were sacrificed at 7 days (AT-1, n = 5 and MatLyLu, n = 8), 10 days (AT-1, n = 13 and MatLyLu, n = 9), 14 days (AT-1, n = 11), 42 days (2 x 10^5^ G cells, n = 7), and at 49 days (2 x 10^3^ G cells, n = 6) after tumor cell injection. Rat prostates injected with RPMI medium were used as tumor-free controls (n = 6). At sacrifice, the prostate and various other organs were removed, weighed, and prepared as described earlier [5–7]. For RNA preparation, G tumors (2 x 10^5^ cells, 42 days, n = 8), AT-1 tumors (2 x 10^3^ cells, 10 days, n = 8), MatLyLu tumors (2 x 10^3^ cells, 10 days, n = 7), and tumor-free controls (RPMI, 10 days, n = 7) were snap-frozen in liquid nitrogen.

### Immunohistochemistry

Tissue from rat or human specimens was immunostained using the Ventana Benchmark Ultra automatic staining system (Ventana Medical Systems Inc., Tucson, AZ). Briefly, 5-μm-thick paraffin sections were pretreated with CC1 for antigen retrieval and stained with primary polyclonal antibodies against HO-1 (ENZO for rat tissue, cat. no. ADI-SPA-895, NY, and ATLAS/Sigma-Aldrich for human tissue, cat. no. HPA000635, St. Louis, MO). Sections were also stained for CD68 (AbD Serotec for rat tissue, cat. no. MCA341, Kidlington, UK, and Dako for human tissue, cat. no. M0814, Älvsjö, Sweden), CD163 (AbD Serotec for rat tissue, cat. no. MCA342R), and factor VIII (Dako cat. no. A0082 for human tissue) as described earlier [6, 7, 10, 11]. Samples were visualized using the ultraView Universal DAB detection Kit. Sections were also stained to visualize cellular Fe deposits using Prussian blue staining [39]. Specificity of the rat HO-1 antibody (ENZO) was examined by pre-incubation of the antibody with a 100-fold excess (w/w) of a recombinant rat HO-1 peptide (ENZO, cat. no. ADI-SPP-730-D). The specificity of the HO-1 antibody used for the human samples (ATLAS) has been validated by the Human Protein Atlas (www.proteinatlas.org).

The volume densities of tumor tissue in the prostate and of various cell types in the tumor-bearing organ (TINT) and in prostate tumors was determined by using a square-lattice mounted in the eyepiece of a light microscope and counting cross sections falling on the measured tissue compartment and reference tissue, as described earlier [5, 6, 10, 11]. The values measured therefore represent the average cell density in tumors and in the benign part of the tumor-bearing prostate lobe.

### RNA preparation and quantitative RT-PCR analysis

Frozen prostate tissues, containing either G, MatLyLu, or AT-1 tumors and the surrounding non-malignant tissue, were sectioned and stained with hematoxylin-eosin to determine tumor location. Using the sections as guidance, frozen tumor tissue and surrounding non-malignant tissue were separated and dissected with a surgical blade as previously described [4]. RNA was extracted using the TRIzol method according to protocol (Invitrogen, Stockholm, Sweden). Total RNA was DNase-treated (DNase 1, Ambion/ThermoFisher Scientific) to remove contaminating DNA, and 0.2 μg was used for synthesis of cDNA using Superscript VILO (Invitrogen) according to the manufacturer’s instructions. The real-time qRT-PCR was performed using the Applied Biosystems 7900HT Real-Time PCR System and the Taqman Gene Expression Assay (Applied Biosystems, Austin, TX). The quantification of mRNA levels was done in a 20-μl reaction volume with 20 ng cDNA per reaction for HO-1 (commercially available primer and probe mix, Rn01536933_m1, Applied Biosystems). Negative controls were run in parallel, and the relative values for each gene were normalized using beta-actin (Rn00667869_m1) as the reference gene and analyzed with Taqman Analysis Software SDS2.4 (Applied Biosystems).

### Patients

Tissue specimens were collected from men who underwent transurethral resection of the prostate (TURP) between 1975 and 1991, and where histological analysis showed the presence of prostate cancer [40]. Local tumor stage was determined by digital rectal examination at the time of surgery and the presence of metastases was evaluated by radionucleotide bone scan. In a pilot study, we noted that HO-1^+^ cells were rare in human prostate tissue and were detected mainly in high-grade tumor tissue. When HO-1^+^ cells were detected, they were found in foci containing several positive cells situated at the border between neoplastic cells and the stroma. To explore this in more detail, we therefore stained sections from 45 Gleason score (GS) 7–10 patients (14 GS 7 and 31 GS 8–10) using an anti-HO-1 antibody (ATLAS, as described above). The number of foci with HO-1^+^ cells was counted and expressed as number of foci per unit area (1.3 mm^2^).

The HO-1 expression in bone metastatic disease was examined by exploring global transcriptome data from a set of untreated (hormone-naïve, n = 10) and castration-resistant (n = 31) human bone metastases that were analyzed in relation to non-malignant (n = 13) and malignant (n = 12) tissue samples from radical prostatectomies using the human HT12-v3 Illumina Beadchip gene expression array (Illumina, San Diego, CA, USA) as previously described in detail [41]. HO-1 protein expression in metastases was analyzed by immunohistochemistry (ATLAS antibody, as described above).

### Statistics

The Mann-Whitney U-test was used for comparison between groups. A p-value <0.05 was considered significant. The Spearman rank correlation coefficient (*R*s) or Kendal-Tau was calculated for correlation studies. Statistical analysis was performed using the statistical software Statistica 12.0 (StatSoft, Tulsa, OK, USA).

## Results

### HO-1 expression in rat prostate tumors and in the surrounding tumor-bearing organ

#### HO-1 mRNA expression in tumor tissue and TINT

We examined HO-1 mRNA expression in rat prostate tumors and in the benign parts of the tumor-bearing prostate lobe (here named TINT). For this purpose, we used three different rat prostate cancer cell lines: (1) the slow-growing and non-metastatic Dunning G (G) cells, (2) the locally aggressive but poorly metastatic AT-1 cells, and (3) the highly aggressive and metastatic MatLyLu cells [5, 38]. The tumor cells were injected into the prostate and difference sizes of each tumor type were obtained either by following them over time (AT-1 and MatLyLu) or by injecting different numbers of cells (G). To enable studies of TINT, all animals were sacrificed when the tumors were still surrounded by normal prostate tissue [5].

**Fig 1:**
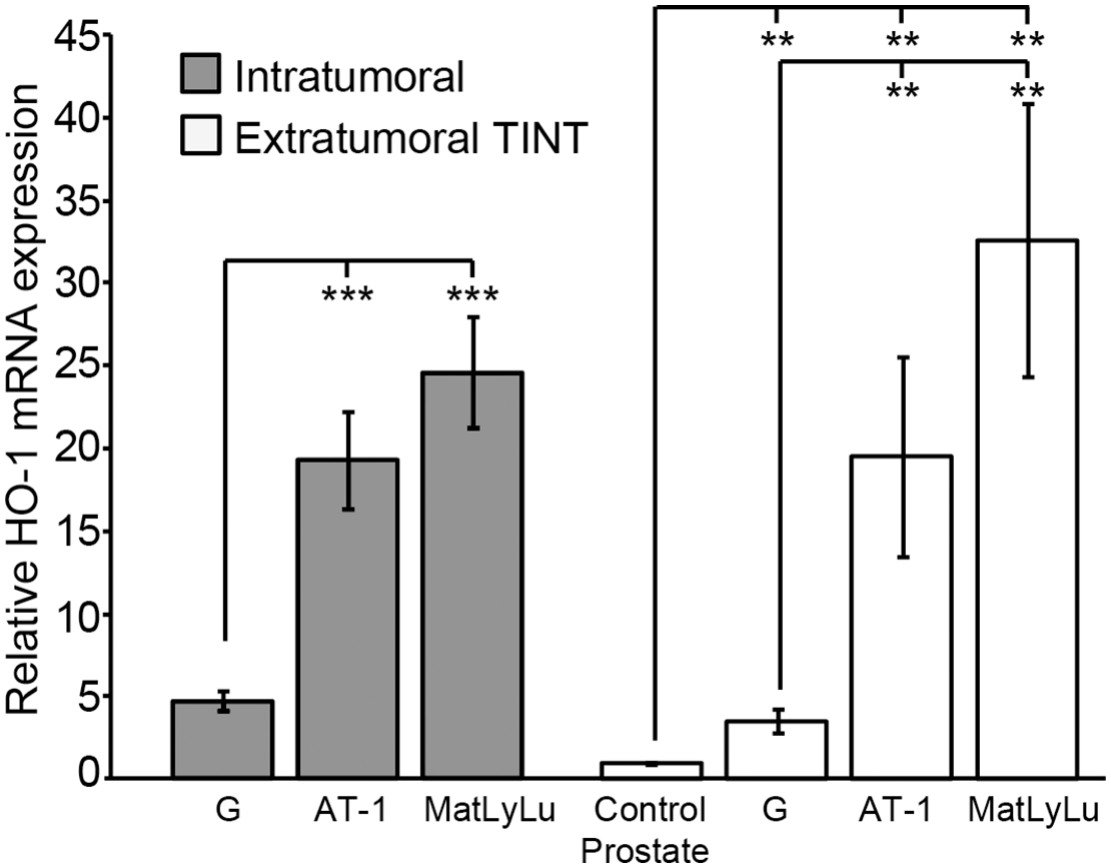
HO-1 mRNA expression in rat prostate tumors and in the surrounding non-malignant prostate tissue (TINT). HO-1 mRNA expression in rat tumors and TINT expressed in relation to the levels in tumor-free control prostate tissue (**p <0.01, ***p < 0.001, n = 7–8 in each group).

In tumor tissue, HO-1 mRNA expression increased with increasing tumor aggressiveness (Fig 1). HO-1 mRNA expression in TINT was increased compared to sham-injected tumor-free control prostate tissue, and was higher in AT-1 and MatLyLu TINT than in G TINT (Fig 1). AT-1 and MatLyLu tumors had markedly higher HO-1 mRNA expression *in vivo* than in the corresponding cell line *in vitro* (data not shown).

#### HO-1 was mainly expressed in macrophages accumulating in TINT and at the tumor border

**Fig 2:**
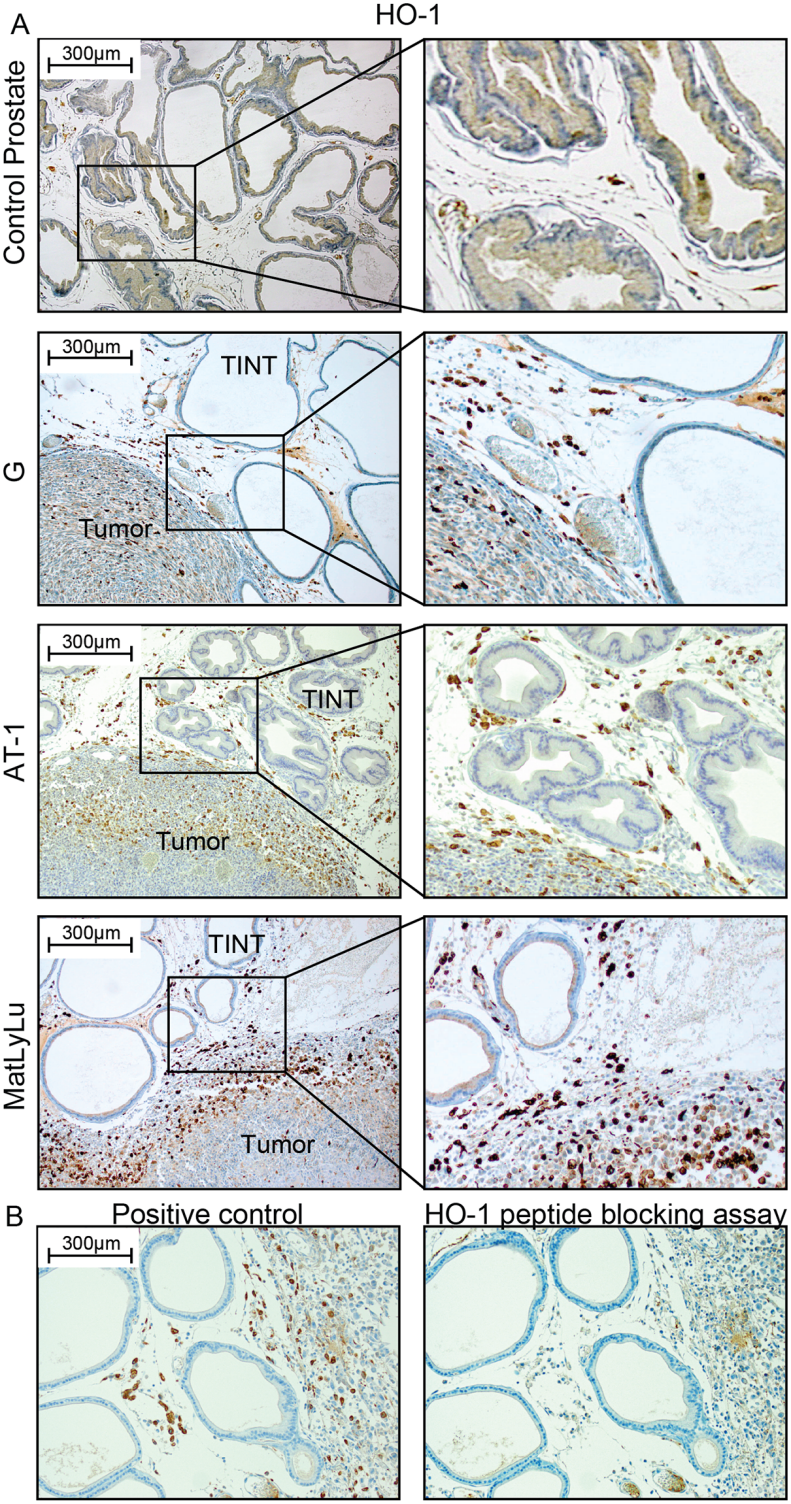
HO-1 protein expression in rat prostate tumors and in the surrounding non-malignant prostate tissue (TINT). (A) Representative sections of control rat prostate tissue and orthotopic rat prostate tumors and TINT stained for HO-1 (brown) (left panel; 100x magnifications, right panel; shows higher magnifications). (B) Antibody specificity control. No staining was seen in sections incubated with primary antibody that had been pre-incubated with an excess of a recombinant rat HO-1 peptide.

Using immunohistochemistry, HO-1 protein expression was examined in the different rat prostate tumor subtypes and in the surrounding normal prostate tissue. Tumor epithelial cells and normal prostate epithelial cells showed limited immune staining of HO-1 (Fig 2). Intense HO-1 staining was observed in infiltrating inflammatory cells, and they were seen in increased numbers in the entire tumor-bearing prostate lobe compared to tumor-free control prostate tissue (Fig 2). HO-1^+^ cells were particularly abundant in TINT that was close to the tumor, and at the tumor border (Fig 2). Conversely, the number of HO-1^+^ cells was low in the more central parts of the tumors, except in areas of necrosis (Fig 2).

**Fig 3:**
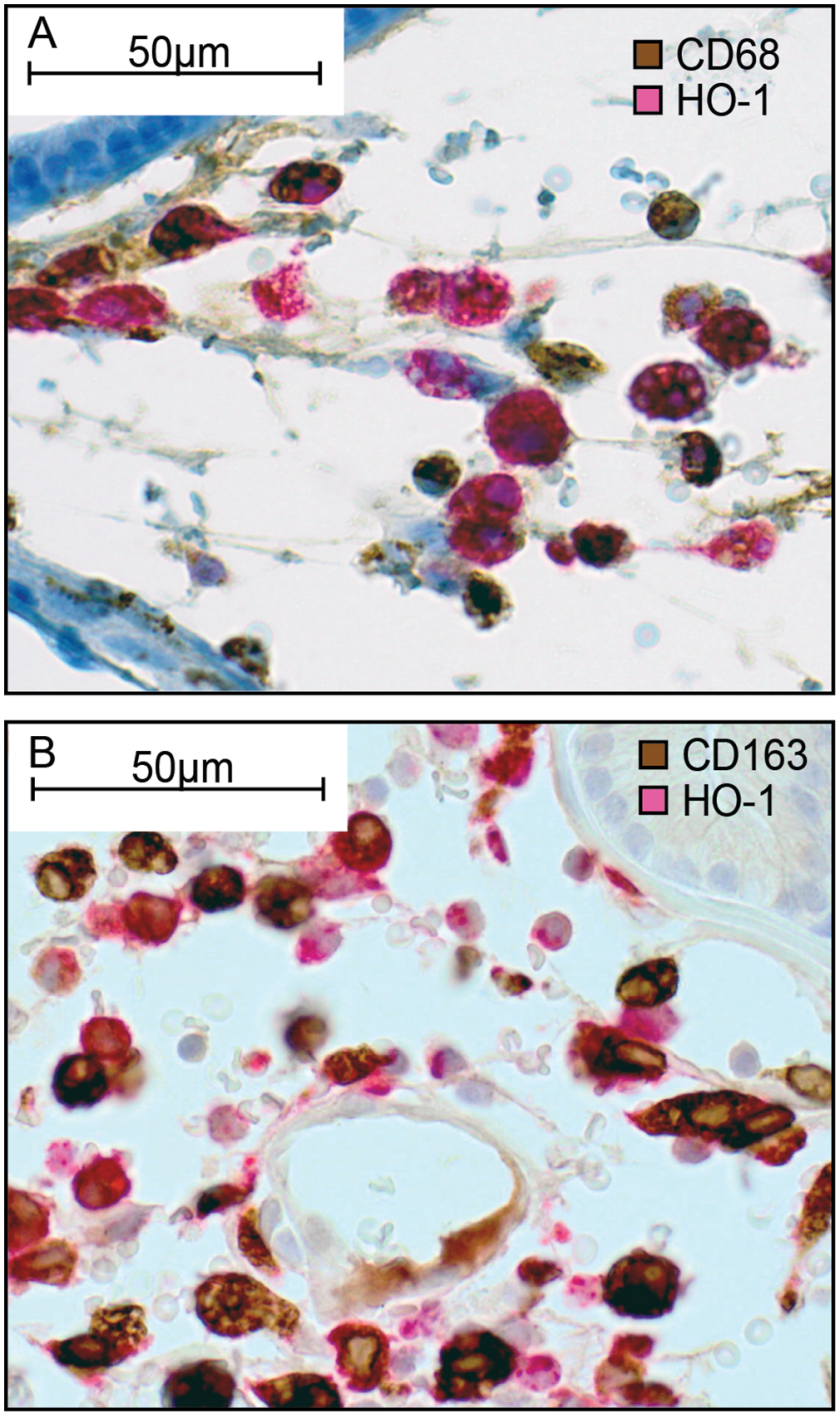
HO-1 expression in macrophages. Representative sections (400x magnifications) of rat prostate MatLyLu TINT double stained for CD68^+^ (brow) and HO-1^+^ (red) cells (A), or double stained for CD163^+^ (brown) and HO-1^+^ (red) cells (B). Most cells stained both red and blown suggesting that most HO-1^+^ cells are macrophages, and in particular of the M2-type (CD163^+^).

HO-1 is normally produced by macrophages [16, 18, 19]. Double-staining showed that HO-1^+^ cells were generally CD68^+^ (a pan-macrophage marker) and CD163^+^ (an M2-macrophage marker) (Fig 3A and 3B). A few cells were HO-1^+^ and CD163^-^ (Fig 3B), suggesting that CD163^+^ and HO-1^+^ macrophages were overlapping population but not identical ones. In TINT and normal control prostate tissue, the density of HO-1^+^ cells (see below) was highly correlated to the density of both CD68^+^ cells (Rs = 0.89, p < 0.05) and CD163^+^ cells (Rs = 0.85, p < 0.05, using our data from Adamo et al. [5]). In AT-1 TINT and MatLyLu TINT, the density of HO-1^+^ cells was slightly lower than the density of CD68^+^ macrophages (the HO-1^+^/CD68^+^ ratio ranged from 0.6 to 1.0), and similar to that of CD163^+^ (the HO-1^+^/CD163^+^ ratio ranged from 0.8 to 1.1) suggesting that HO-1 was expressed in a major subset of TINT macrophages. HO-1 expression in other inflammatory cells, that accumulate in considerably lower numbers than macrophages (Adamo et al. [5]), can however not be excluded. In tumors, the average density of HO-1^+^ cells was considerably lower than that of CD68^+^ macrophages (ratio HO-1^+^ / CD68^+^ ranged from 0.05 to 0.4) suggesting that intratumoral macrophages are generally not HO-1^+^.

#### HO-1+ cells in TINT were related to tumor size and tumor aggressiveness

**Fig 4:**
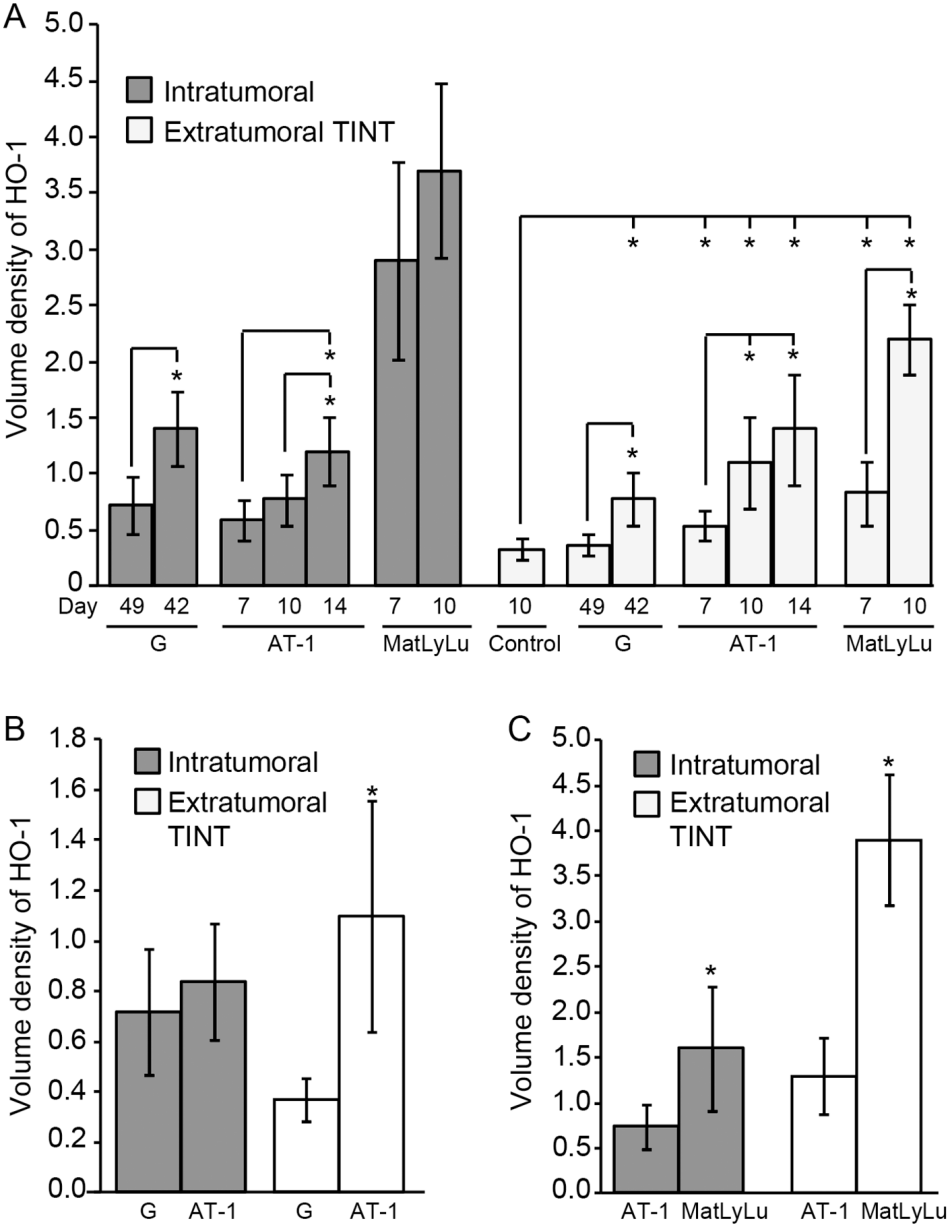
Volume density of HO-1^+^ cells in rat prostate tumors and in the surrounding non-malignant prostate tissue (TINT). (A) Volume density of HO-1^+^ cells–at different time points and tumor sizes (mean tumor weight mg +/- SD)–in G tumors (day 49 (small); 49 +/- 21 mg and day 42 (large); 250 +/- 164 mg), in AT-1 tumors (day 7; 15 +/- 4.5 mg, day 10; 71 +/- 48 mg, and day 14; 458 +/- 406 mg), and in MatLyLu tumors (day 7; 35 +/- 24 mg and day 10; 140 +/- 111 mg) and in TINT (*p < 0.05, n = 5–13 in each group). (B) Volume density of HO-1+ cells in tumor and TINT of slow growing G tumors (n = 6) compared to aggressive AT-1 tumors (n = 9, *p <0.05) of similar sizes (49 +/- 21 and 48 +/- 33 mg, respectively). (C) Volume density of HO-1+ cells in tumor and TINT of non-metastatic AT-1 tumors (n = 8) compared to metastatic MatLyLu tumors (n = 7, *p < 0.05) of similar sizes (97 +/- 39 and 90 +/- 47 mg, respectively).

In all three tumor types, the volume density of HO-1^+^ cells in TINT increased significantly with tumor size, and compared to tumor-free control tissue (Fig 4A). Furthermore, the density of HO-1^+^ cells in TINT increased with tumor aggressiveness, suggesting that fast-growing and metastatic tumors attract more HO-1^+^ macrophages (as well as CD68+ and CD163+ macrophages, as reported earlier [5]) to the tumor-bearing organ than slow-growing and non-metastatic tumor variants. The intratumoral density of HO-1^+^ cells also increased somewhat with tumor size, and was highest in the aggressive and metastatic MatLyLu tumors (Fig 4A).

In order to compare HO-1^+^ cell accumulation in fast-growing AT-1 tumors (day 10) and slow-growing G tumors (day 49) of similar sizes (mean tumor weight +/- SD: 49 +/- 21 mg and 48 +/- 33 mg, respectively), we excluded some of the smallest G tumors and largest AT-1 tumors in our dataset. This showed that AT-1 tumors were more effective than G tumors in attracting HO-1^+^ cells into the TINT, but that they were equally effective in attracting HO-1^+^ cells into the tumor (Fig 4B). We also compared poorly metastatic AT-1 tumors (day 10) and highly metastatic MatLyLu tumors (day 10) of similar sizes (97 +/- 39 mg and 90 +/- 47 mg, respectively) by excluding some of the largest MatLyLu tumors and smallest AT-1 tumors. This showed that MatLyLu tumors were more effective in attracting HO-1^+^ cells into the tumor and into the TINT than AT-1 tumors (Fig 4C).

### HO-1^+^ and iron-containing macrophages accumulate at sites of tumor-induced tissue bleeding

**Fig 5:**
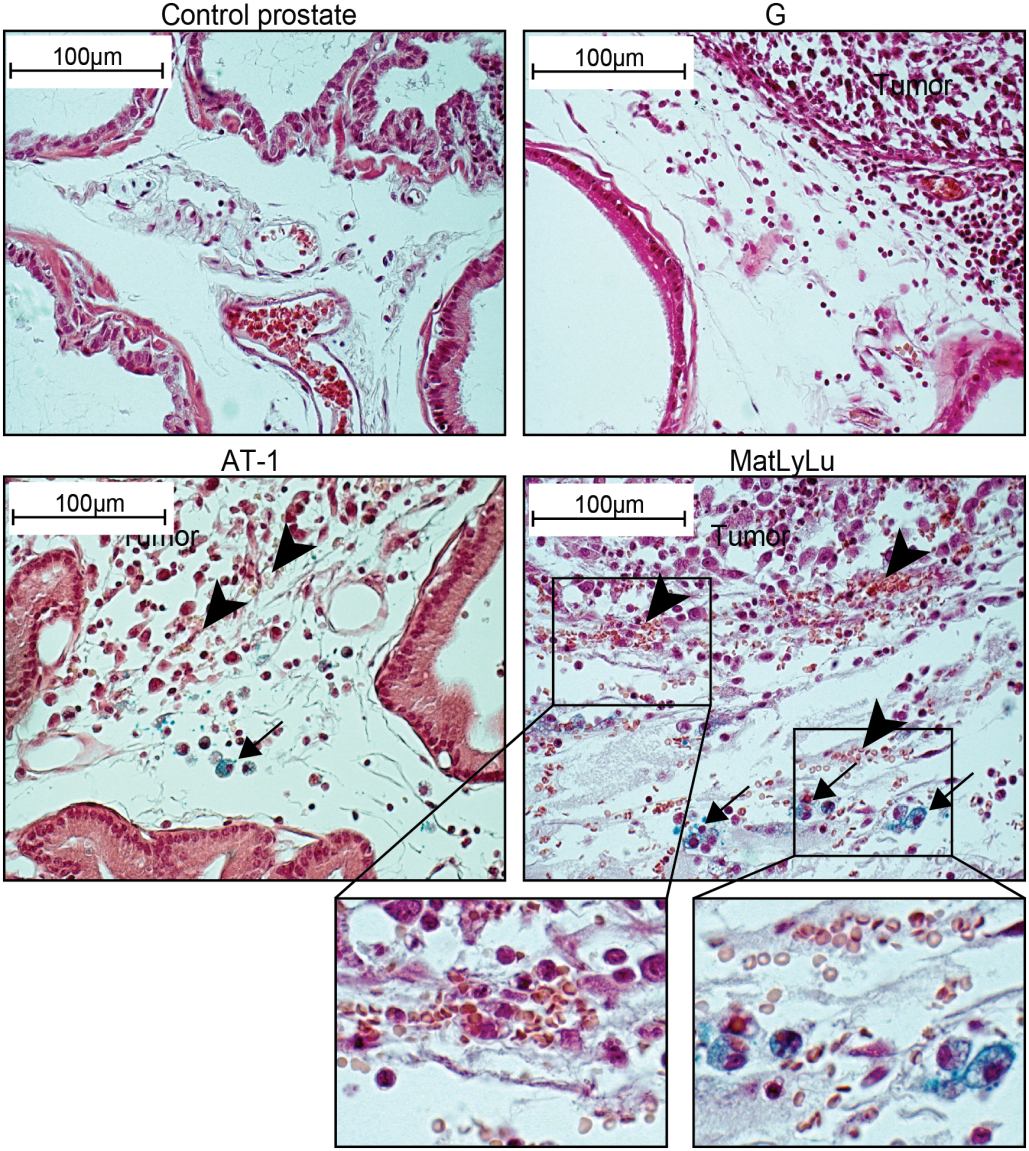
Extravasated erythrocytes and intracellular iron in rat prostate tumors and in the surrounding non-malignant prostate tissue (TINT). Prussian-blue stained sections showing different densities of extravasated erythrocytes (arrowheads) and intracellular iron in macrophages (blue, arrows) in control prostate tissue and at the tumor border of G, AT-1 and MatLyLu tumors (original magnification 400x). Extravasated erythrocytes and iron^+^ macrophages were not found in control prostates and were uncommon in G TINT. Bleeding was common at the tumor border of AT-1 and MatLyLu tumors and these tumors also contained iron^+^ macrophages (insert shows bleeding and iron+ cells at higher magnifications).

One function of HO-1 is to metabolize heme from hemoglobin. HO-1 expression is therefore upregulated at sites with increased bio-availability of heme [42]. Extravasated erythrocytes were often seen at the border zone between the tumor and the surrounding normal prostate tissue (Fig 5), thus corresponding to the same site where HO-1^+^ cells were most abundant (Fig 2). The densities of extravasated erythrocytes and HO-1^+^ macrophages were low at the tumor border of G tumors, and they increased in the more aggressive AT-1 and MatLyLu tumors (Fig 5). Inside tumors, bleeding was seen occasionally in necrotic areas but rarely in viable tumor tissue, which might explain why HO-1^+^ cells were found in necrotic areas. AT-1 and MatLyLu tumors often contained necrotic areas while the slow-growing G tumors did not (data not shown).

In Prussian blue-stained sections, intracellular iron-deposits were seen in macrophages in MatLyLu TINT and to some extent also in AT-1 TINT, but Fe-containing macrophages were rare in G TINT and they were not detected in 10-day sham-injected control prostate (Fig 5). This suggests that bleeding and accumulation of Fe in macrophages may be associated with high HO-1 expression.

### HO-1 expression in primary and metastatic prostate tumors in patients

#### HO-1+ macrophages were found at the invasive front of the tumor and were associated with high tumor grade and presence of bone metastases

HO-1 expression was examined in human primary prostate tumors and in bone metastases using immunohistochemistry. Human liver and lung tissues were used as positive controls, and showed numerous HO-1^+^ macrophages (Kupfer cells and alveolar macrophages) (data not shown). In line with the protein atlas (www.proteinatlas.org), urothelial cells were moderately positive and thus served as an internal positive control.

**Fig 6:**
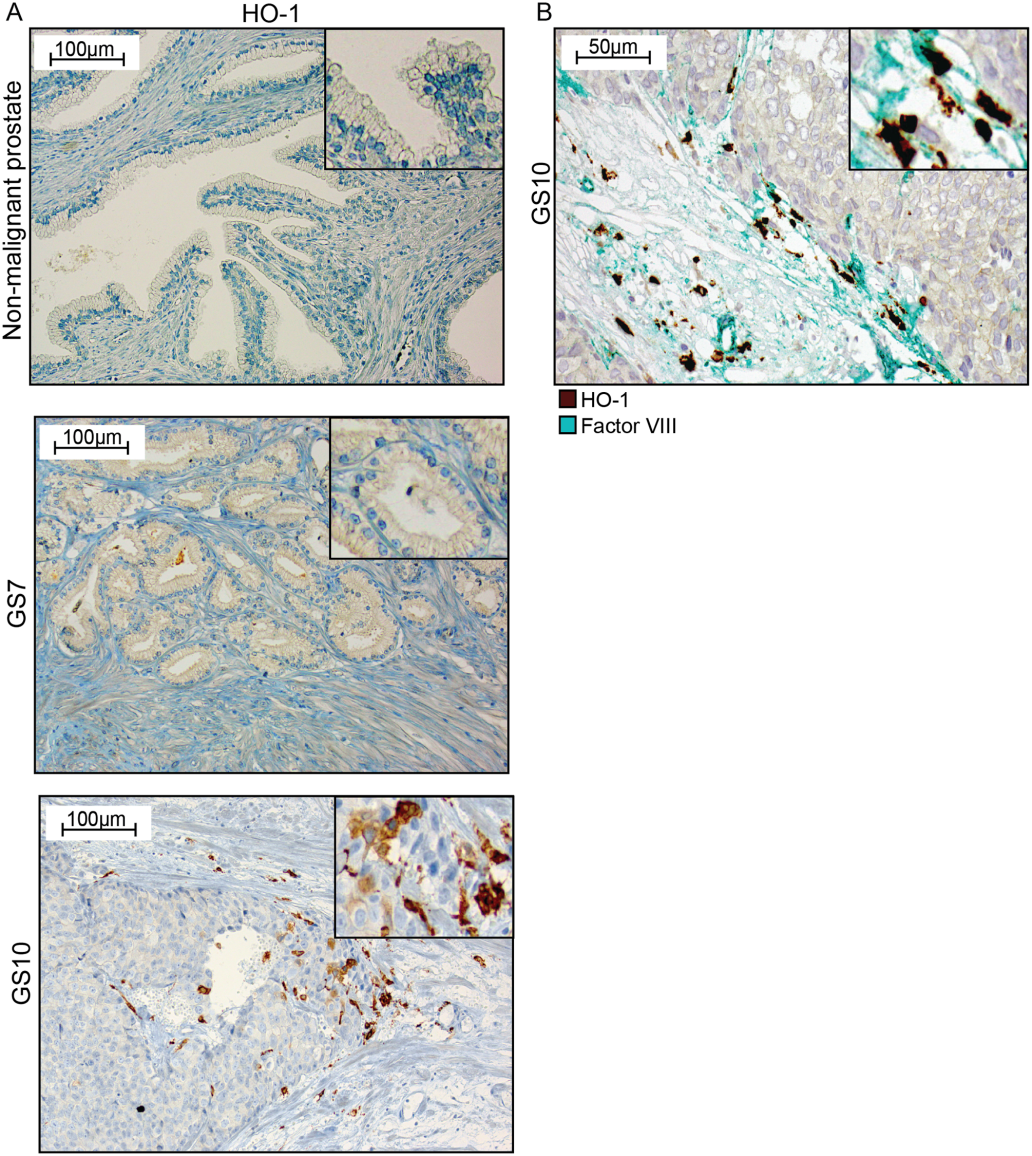
HO-1 expression in human primary prostate tumors. (A) Representative sections of HO-1 staining (brown) in non-malignant prostate tissue, in a Gleason score (GS) 7 primary prostate tumor, and in a GS 10 primary prostate tumor (200x magnifications, inserts show higher magnifications). (B) A GS 10 tumor double stained for factor VIII positive blood vessels (green) and HO-1^+^ cells (brown) (400x magnifications, and insert at higher magnifications).

In non-malignant prostate tissue in prostate cancer patients, HO-1 staining was observed only in a few cells in the stroma, and normal glandular epithelial cells were generally unstained (Fig 6A). In primary prostate tumors, epithelial cells were generally unstained (Fig 6A). Although the tumor stroma was generally devoid of HO-1^+^ cells, foci of HO-1^+^ cells could occasionally be observed at the border between high-grade tumor epithelial cell nests and the surrounding stroma (Fig 6A). These HO-1^+^ cells were CD68^+^ (data not shown). However, the number of HO-1^+^ macrophages appeared to be considerably lower than previously reported for CD68^+^ [43], S100A9^+^ [36], and CD163^+^ macrophages [35]. In areas with HO-1^+^ cells, blood vessels were often seen close to tumor cells–suggesting a possible local site for vascular invasion and erythrocyte leakage (Fig 6B).

Foci with HO-1^+^ cells were uncommon in GS 7 tumors (0.14 +/- 0.36 per unit area, n = 14), but they were significantly increased in GS 8–10 tumors (0.90 +/- 1.2 foci per unit area, mean +/- SD, n = 31, p = 0.03, Mann-Whitney U-test). The number of foci with HO-1^+^ cells was correlated to GS (Kendall-Tau = 0.31, p < 0.05). When we compared the number of foci with HO-1^+^ macrophages in GS 7–10 tumors with metastases (n = 14) to the number of GS 7–10 tumors without metastases (n = 31) at diagnosis, it was 0.48 +/- 1.0 per unit area in non-metastatic tumors and 1.1 +/- 1.2 in metastatic tumors (p = 0.05). There was a correlation between the number of foci with HO-1^+^ cells and the presence of metastases (Kendal Tau = 0.28, p < 0.05).

#### HO-1 levels were high in prostate cancer bone-metastases

**Fig 7:**
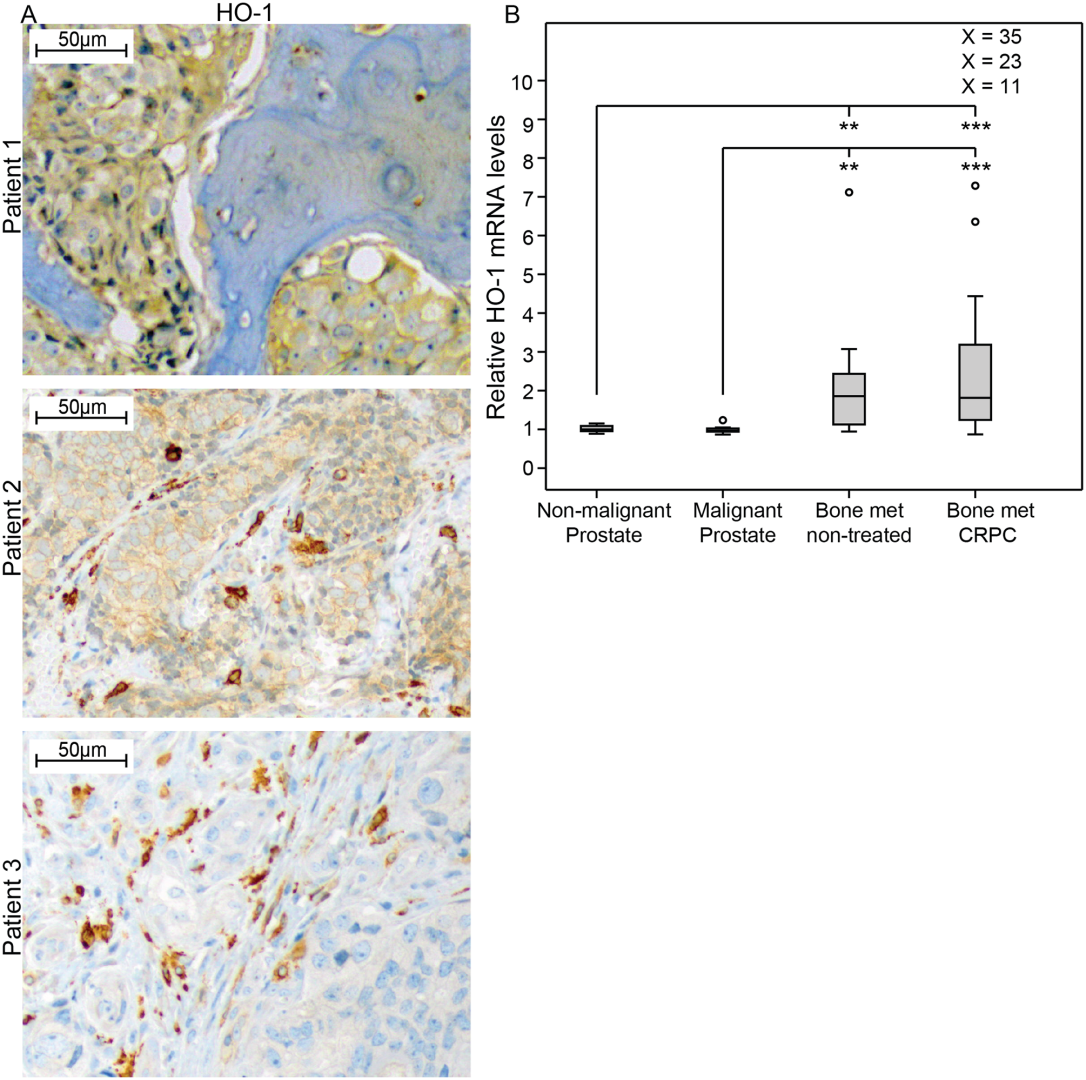
HO-1 expression in human prostate bone metastases. (A) Representative sections of HO-1 staining (brown) in bone metastases, showing positive staining in tumor epithelial cells (patient 1), in tumor epithelial cells and in macrophages (patient 2), and in macrophages (patient 3) (200x magnifications). (B) Relative HO-1 mRNA expression in non-malignant prostate tissue samples (TINT) (n = 13), malignant prostate tissue samples (n = 12), and bone metastasis tissue samples obtained from previously untreated (n = 10) or castration resistant prostate cancer (CRPC, n = 31) patients, according to data from whole genome cDNA array profiling using the Illumina HumanHT-12 v3 Expression BeadChip [41]. Extreme values are indicated with an open circle, but for values out of the figure scale the relative expression value is given. **p <0.01, ***p<0.001.

Having found that HO-1 could possibly be related to tumor aggressiveness and metastasis, we analyzed HO-1 in prostate cancer bone metastases. Immunohistochemistry showed that HO-1^+^ cells, presumably macrophages, were abundant in normal bone marrow, and in bone metastases (Fig 7A). In the metastases, as in primary tumors, HO-1^+^ macrophages were seen at the tumor cell-stroma interface and only a few were present inside the metastases (Fig 7A). The density of HO-1^+^ macrophages per unit area varied between metastases, but was considerably higher (2- to 48-fold) than the average value in primary tumors with metastases. In contrast to primary tumors, in about two-thirds of the metastases the tumor epithelial cells were moderately stained for HO-1 (Fig 7A). This tumor epithelial cell staining was mainly seen in the cytoplasm and not in the nucleus.

We have previously examined the global transcriptome in normal prostate tissue adjacent to tumor, in primary prostate cancer, and in bone metastases [41]. In line with the immunohistochemistry data, HO-1 mRNA levels were similarly low in normal and primary tumor tissue, but significantly higher in bone metastases (Fig 7B). There was no significant difference in HO-1 mRNA levels between castration-resistant prostate cancer (CRPC) and bone metastases from previously untreated PC (Fig 7B). Using our previously published transcriptome data [41], we found that the inter-patient variation in HO-1 mRNA levels in metastases was large and CRPC cases showed an inverse correlation to AR mRNA levels (Rs = -0.41, p = 0.022, n = 31) while being positively correlated to mRNA levels for CD68 (Rs = 0.70, p = 0.000013, n = 31) and CD163 (Rs = 0.53, p = 0.002, n = 31), but not iNOS (Rs = -0.17, p = 0.38, n = 31), indicating HO-1 expression in M2 macrophages in human PC bone metastases with less AR activity.

## Discussion

We previously reported that CD68^+^ macrophages accumulate in the benign parts of tumor-bearing prostate lobes (the TINT), that depletion of these cells inhibited tumor growth [10, 11], and that HO-1 (presumably a tumor-stimulating factor [21]) was markedly upregulated in rat TINT [4]. In this study, we therefore tried to investigate possible roles of HO-1 in prostate tumors and in TINT in more detail.

HO-1 was expressed in macrophages that accumulated in the entire tumor-bearing prostate lobe, and in particular at the border zone of rat prostate tumors. Fast-growing and metastatic tumors attracted more HO-1^+^ cells to the tumor-bearing organ than slow-growing and non-metastatic tumor variants. The underlying mechanisms for this are unknown, but HO-1 is an inducible enzyme, and one key factor that increases its expression is heme [42]. Heme is derived from hemoglobin, and HO-1 expression is therefore upregulated at sites of tissue injury and bleeding. Microscopic foci of bleeding and Fe-containing macrophages were particularly common at the invasive front of aggressive MatLyLu tumors, but rare at the borders of the more indolent G tumors. Increased HO-1 expression in the peripheral parts of tumors, in necrotic tumor tissue, and in the surrounding normal prostate could therefore be caused by increased availability of heme. Bleeding may also explain why the gene expression pattern in TINT was characterized by activation of coagulation, and why expression of tissue factor (the protein that starts coagulation) was increased [4]. HO-1 itself may also promote coagulation [44].

Not all HO-1^+^ cells in the prostate were, however, positive for the CD163 receptor, the receptor for heme uptake [42], suggesting that additional factors may have stimulated the HO-1 expression. In other organs, HO-1 was induced by cytokines, interleukins, M-CSF, ROS, NO, TGF-beta, PDGF, prostaglandins, insulin, LPS, adipokines, and tissue hypoxia [21]. Several of these factors, including hypoxia, are increased in prostate tumors and in TINT [5]. Exosomes from MatLyLu and G tumors increased the expression of HO-1 in rat monocytes *in vitro*, and MatLyLu exosomes were more potent than G exosomes (Halin Bergström et al. unpublished). Multiple mechanisms such as bleeding, hypoxia, and signals from tumor epithelial cells could therefore explain the increased expression of HO-1 in macrophages in TINT and in prostate tumors.

Further studies are required to understand the functional role of HO-1^+^ macrophages in prostate cancer. Macrophages deliver Fe to cancer cells in other tumor types [45, 46], and accumulation of Fe in prostate cancer cells was found to be associated with poor patient outcome [47]. It is therefore possible that bleeding around aggressive tumors, with accumulation of heme-degrading M2-macrophages, facilitates iron delivery to neoplastic cells at the invasive front. Inhibition of HO-1 has retarded tumor growth in other experimental models of prostate cancer [22, 24] and, importantly, tumor growth and lung metastasis were found to be reduced in animals with selective depletion of HO-1 in macrophages [33]. In a breast cancer model, inhibition of HO-1 in macrophages polarized tumor-associated macrophages towards a tumor-inhibiting M1 phenotype [48]. Here we show that aggressive and metastatic tumors attracted more HO-1^+^ macrophages to the tumor-bearing organ than more indolent tumors, and that the main site of HO-1 action is probably not within but outside tumors. Altogether, these results suggest that HO-1 expressing macrophages accumulating outside and at the invasive zone of prostate tumors stimulate tumor growth and metastasis.

In comparison to aggressive rat tumors, the number of HO-1 expressing cells at the tumor-invasive zone was found to be low in most prostate cancer patients–and HO-1 mRNA levels were low in primary prostate tumors and in adjacent non-malignant prostate tissue. Foci with HO-1^+^ macrophages were, however, present in the periphery of high GS tumors, and the number of such foci was correlated to the presence of bone metastases. The correlation was, however, weak and more studies are needed to investigate whether scattered peritumoral foci of HO-1^+^ macrophages influence primary tumor aggressiveness and metastatic spread.

HO-1 mRNA levels in prostate cancer bone metastases were considerably higher than in primary tumors. HO-1^+^ macrophages were common at the invasive zone of human bone metastases, suggesting that HO-1 expressing macrophages may play a more significant role in metastases than in primary tumors. In bone metastases, HO-1 was detected in tumor epithelial cells, although nuclear staining was rare. In line with this, studies in experimental models have shown that factors in the bone microenvironment upregulate HO-1 in prostate cancer epithelial cells [49]. Interestingly, HO-1 mRNA levels in bone metastases were correlated to the expression levels of macrophage markers such as CD68 and CD163, and inversely correlated to expression of androgen receptors [41, 50]. As expression levels of AR can be used to characterize different subgroups of castration-resistant bone metastases probably needing different types of treatments [41, 50], the roles of extratumoral macrophages, HO-1, iron metabolism, and coagulation in prostate cancer bone metastases should be explored in more detail.

Several studies using immunohistochemistry and quantitative RT-PCR have shown that HO-1 is expressed in prostate cancer cells, particularly in tumor cell nuclei, and this was found to be associated with high GS and poor outcome [22, 24–28]. In contrast, experimental overexpression of HO-1 in tumor cells retarded experimental prostate cancer growth [30–32]. In the present study, HO-1 expression was observed in metastatic prostate cancer cells but was rarely observed in primary tumor or TINT epithelial cells, and seldom in the nuclei. The reason for this discrepancy is unknown, but it could be related to the antibodies used. As we detected HO-1 in macrophages, a well-established site for HO-1 synthesis, our approach was suitable to explore the potential role of HO-1 in macrophages–whereas other antibodies may be more useful in exploring the role of HO-1 expression in various compartments of tumor epithelial cells. Whatever the role of tumor cell-derived HO-1, our results suggest that extratumoral HO-1 expressing macrophages may be involved in prostate cancer aggressiveness and metastatic capacity, and that their role in bone metastases in particular should be investigated in more detail.
